# The Metabolic Impact of Two Different Parenteral Nutrition Lipid Emulsions in Children after Hematopoietic Stem Cell Transplantation: A Lipidomics Investigation

**DOI:** 10.3390/ijms23073667

**Published:** 2022-03-27

**Authors:** Oscar Daniel Rangel-Huerta, María José de la Torre-Aguilar, María Dolores Mesa, Katherine Flores-Rojas, Juan Luis Pérez-Navero, María Auxiliadora Baena-Gómez, Angel Gil, Mercedes Gil-Campos

**Affiliations:** 1 Section of Chemistry and Toxinology, Norwegian Veterinary Institute, P.O. Box 64, N-1431 Ås, Norway; oscar.daniel.rangel.huerta@vetinst.no; 2 Department of Pediatrics, Unit of Pediatric Research, Reina Sofia University Hospital, Maimonides Institute of Biomedical Research of Cordoba (IMIBIC), University of Córdoba, Avda Menéndez Pidal s/n, 14004 Cordoba, Spain; delatorremj4@gmail.com (M.J.d.l.T.-A.); katherine1.flores@gmail.com (K.F.-R.); juanpereznavero@hotmail.com (J.L.P.-N.); mabaenagomez@gmail.com (M.A.B.-G.); mercedes_gil_campos@yahoo.es (M.G.-C.); 3 Department of Biochemistry and Molecular Biology II, Institute of Nutrition and Food Technology, Center of Biomedical Research, University of Granada, Avda. del Conocimiento s/n, 18016 Armilla, Spain; mdmesa@ugr.es; 4 Instituto de Investigación Biosanitaria ibs.Granada, 18012 Granada, Spain; 5 CIBEROBN (Physiopathology of Obesity and Nutrition), Institute of Health Carlos III (ISCIII), 28029 Madrid, Spain

**Keywords:** bone marrow transplantation, fat emulsions, intravenous, fatty acids, omega-3, hematopoietic stem cell transplantation, lipidomics, parenteral nutrition

## Abstract

Hematopoietic stem cell transplantation (HSCT) involves the infusion of either bone marrow or blood cells preceded by toxic chemotherapy. However, there is little knowledge about the clinical benefits of parenteral nutrition (PN) in patients receiving high-dose chemotherapy during HSCT. We investigated the lipidomic profile of plasma and the targeted fatty acid profiles of plasma and erythrocytes in children after HSCT using PN with either a fish oil-based lipid emulsion or a classic soybean oil emulsion. An untargeted liquid chromatography high-resolution mass spectrometry platform connected with a novel in silico annotation algorithm was utilized to determine the most relevant chemical subclasses affected. In addition, we explored the interrelation between the lipidomics profile in plasma, the targeted fatty acid profile in plasma and erythrocytes, several biomarkers of inflammation, and antioxidant defense using an innovative data integration analysis based on Latent Components. We observed that the fish oil-based lipid emulsion had an impact in several lipid subclasses, mainly glycerophosphocholines (PC), glycerophosphoserines (PS), glycerophosphoethanolamines (PE), oxidized PE (O-PE), 1-alkyl,2-acyl PS, lysophosphatidylethanolamines (LPE), oxidized PS (O-PS) and dicarboxylic acids. In contrast, the classic soybean oil emulsion did not. Several connections across the different blocks of data were found and aid in interpreting the impact of the lipid emulsions on metabolic health.

## 1. Introduction

Hematopoietic stem cell transplantation (HSCT) is currently the standard of care for many malignant and nonmalignant blood diseases. HSCT involves the infusion of either bone marrow, peripheral blood or cord blood as a stem cell source preceded by toxic chemotherapy. The conditioning regimen decreases the tumor burden and maximizes the donor cells’ capability to engraft successfully by suppressing the patient’s immune system [1]. This aggressive chemotherapy affects the digestive system and limits oral food intake. Thus, a majority of patients undergoing HSCT suffer from severe mucositis and enteritis due to cytotoxic therapy and immune dysregulation, resulting in prolonged decreased oral intake, nausea, vomiting, and diarrhea. In this regard, nutrition support is often required during HSCT, given the gastrointestinal toxicity that frequently precludes adequate protein-calorie intake [2]. Parenteral nutrition (PN) is often given to patients to maintain their nutritional status during the peritransplant period, and it is reserved for those patients who are unable to tolerate enteral feedings [3,4]. However, the clinical benefits of PN in patients receiving high-dose chemotherapy during HSCT are unknown [5,6]. An integrative review on the efficacy of enteral nutrition and PN for meeting the nutrition and energy needs of pediatric patients following HSCT was published in 2019 [7]. More recently, the Pediatric Diseases Working Party (PDWP) of the European Society for Blood and Marrow Transplantation (EBMT) reported the resulting consensus on the management of sinusoidal obstructive syndrome, mucositis, enteral and parenteral nutrition, iron overload, and emesis during HSCT [8], as well as on prevention of infections [9]. Moreover, early and customized nutritional intervention may be optimal for all patients who undergo HSCT to ameliorate body weight loss associated with nutrition-related adverse events [10].

Previous studies have demonstrated that the use of lipid-based PN after allogeneic bone marrow transplantation is associated with a lower incidence of lethal acute graft-versus-host-disease (aGvHD) and hyperglycemia, without negatively affecting the time to engraftment of infused cells [11,12]. Those studies suggested that the intravenously administered lipids might have influenced the severity of disease by modulation of immune response and synthesis of eicosanoids that participate in the pathogenesis of aGvHD.

Traditionally, the lipids used in artificial nutrition are based on vegetable oils, such as soybean oil (SO), which provide the essential fatty acids (FA) linoleic (LA, 18:2 ω-6) and α-linolenic acid (LNA, 18:3 ω-3) in relatively high amounts. These emulsions have raised concerns because of their potential adverse effects involving oxidative stress, inflammation, and immune response, probably due to the excess of unsaturated FAs [13]. In recent years, fish oil (FO) in lipid emulsions has been introduced as a component of PN. These emulsions contain eicosapentaenoic acid (EPA, 20:5 ω-3) and docosahexaenoic acid (DHA, 22:6 ω-3), which modulate the synthesis of eicosanoids, the activity of nuclear receptors and nuclear transcription factors, and the production of resolvins, protectins, and maresins, with recognized anti-inflammatory and immunomodulatory effects in critically ill patients [14]. Therefore, they have been associated with less hepatic toxicity and lower levels of low-density lipoprotein triacylglycerols and C-reactive protein compared to soybean lipid emulsions in postoperative patients [15]. They are likely to reduce infections, the length of hospital stays, and liver dysfunction without influencing mortality and may be a safe and preferable choice in post-surgery patients [16].

Several studies have revised the effects of PN in children with HSCT [3,4,7]. However, only a few addressed the question of the potential effects of enriched FO lipid emulsions compared with standard lipid emulsions [17,18,19]. We have previously shown that PN containing SO or long-chain polyunsaturated (LC-PUFA) ω-3 FAs enriched emulsions for ten days is safe for children [17]. FO-containing emulsion in long-term PN increases the levels of plasma ω-3 LC-PUFA and decreases those of arachidonic acid (20:4 ω-6) [17] and can improve the antioxidant profile by increasing levels of α-tocopherol in children who have a high risk of suffering oxidative stress and metabolic disorders [18]. In addition, previous findings of our group suggest that different lipid emulsions in PN administered to children undergoing HSCT for a short period do not induce significant inflammatory changes. However, ω-3 LC-PUFA-supplemented PN for more than 21 days may modulate the inflammatory response [19].

Lipids are essential metabolites engaged in several cellular functions; thus, they directly monitor cellular metabolic status. Within the omics sciences context, the complete lipid profile and content in a cell is called lipidome, and the study itself lipidomics. The lipids classification comprises classes and subclasses depending on the head group and the linkage type between the head group and aliphatic chains [20]. A comprehensive work reviewing their functionality can be consulted elsewhere [21]. Some studies carried out in adults with HSCT have shown an altered metabolic profile caused both by the disease and its immunosuppressive treatment [22], and several plasma biomarkers have been claimed to help predict the risk of aGvHD in clinical settings [23,24,25,26]. However, no metabolomic nor lipidomics approaches have been reported in children with HSCT. Therefore, the present study aimed to investigate the plasmatic lipidomics profile in children after HSCT using an FO lipid emulsion and compare the results with those for the classic SO emulsion used in PN. In addition, we investigated the interrelation between the lipidomics profile in plasma, the targeted FA profile in plasma and erythrocytes, several biomarkers of inflammation, and antioxidant defense. The investigated children with HSCT were the same that served for previous publications [17,18,19].

## 2. Results

### 2.1. General Characteristics of the Patients

Table 1 presents a summary of demographics and clinical complications in the population here included. All the patients presented mucositis without significant differences in the degrees of severity (*p* = 0.121). The days of fever post HSTC transplantation were similar in both groups (*p* = 0.204). No patient presented renal pathology or development of hepatic veno-occlusive disease (VOD). Excluding the two patients who received an autologous transplant and therefore cannot be affected by aGvHD, three patients in the SO-based parenteral lipid emulsion (SOPLE) developed GvHD (75%) versus two patients (50%) in the FO-based parenteral lipid emulsion (FOPLE) group (*p* = 0.467). No patient died during admission, and there was a 100-day survival rate of 100% in both groups.

PN was established to prevent or correct the adverse effects of malnutrition and was generally started in these patients when the intake was less than 2/3 of the basal energy needs. It could not be administered enterally, and its need was anticipated by a period greater than 5 to 7 days. In our sample, it began within a range of 1 to 3 days after BMT infusion: PN was administered through a central venous catheter, which all these patients had implanted prior to BMT. Withdrawal of PN was performed when the pathology that caused its onset improved, generally mucositis, and when oral intake reaches 2/3 of the estimated nutritional requirements.

### 2.2. Lipidomics Results

**Figure 1:**
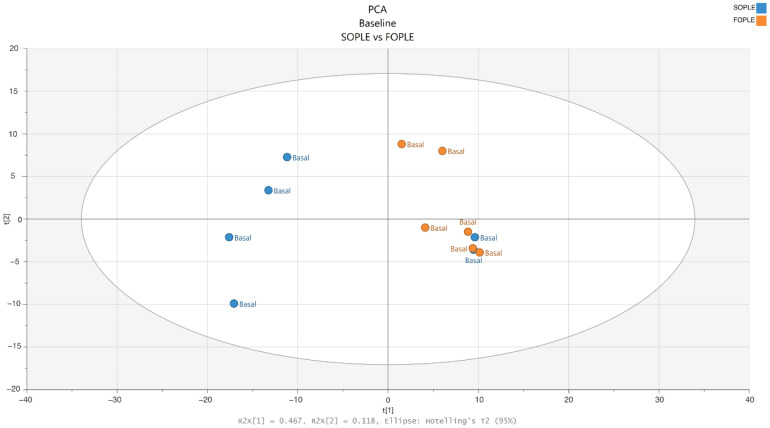
PCA scores plot of the model, including samples from SO-based parenteral lipid emulsion (SOPLE) and FO-based (FOPLE) parenteral lipid emulsion groups at baseline.

Only samples from the entire cohort were included from six subjects following PN with SOPLE and six with FOPLE for the lipidomics analysis. After running the lipidomics and preprocessing the obtained raw data, we detected 612 features in positive mode and 1038 in negative mode. The feature-clustering function identified 235 clusters of two or more features. Thus, the final analysis included 1005 variables corresponding to single features or clusters of features. A principal component analysis (PCA) was used for an initial exploration to assess the quality of the lipidomics data and to detect the presence of outliers (Figure 1). The PCA revealed no outliers, and thus, all the samples were included for further analysis.

#### 2.2.1. Differences at Baseline in the Lipidomics Profile

An orthogonal partial least squares discriminant analysis (OPLS-DA) was implemented to detect if the groups showed a significant difference at baseline. The OPLS-DA is a multivariate supervised model that discriminates between groups, as it tries to maximize the difference among classes using the class as a reference. In this case, the model comparing both groups at baseline was of poor quality, as shown by the low Q_2_ and R_2_ (0.423 and 0.326, respectively) and a not significant CV-ANOVA. Thus, we discarded systematic differences between groups before the intervention. Additionally, we ran a *t*-test among groups, observing no significant differences in any detected metabolites at baseline.

#### 2.2.2. Differences after the Intervention in the Lipidomics Profile

The next step was to detect any differences between the groups associated with the intervention. We utilized an analysis of variance multiblock partial least squares (AMOPLS) for such a purpose. Table 2 summarized the AMOPLS output and revealed that treatment and treatment x time interaction’s main effects were significant as represented by the RSR and R_2_Y *p*-values (*p* ≤ 0.05). It is relevant to mention that the residuals accounted for 75% of the total observed variability. This is a high percentage and shows that other analytical or biological factors might be more relevant in the variation than those here studied.

According to those results, it is necessary to analyze each group’s data independently. Then, two independent OPLS-DA models were built to investigate the differences between the initial and the final samples on each group. On the one hand, it was not possible to generate a significant model for the FOPLE group. On the other hand, the model corresponding to the SOPLE group appeared to be significant (*p* = 0.04). However, when the permutation test plot (Supplementary Figure S1) was inspected, it revealed that the model might be spurious. This can be confirmed by the relatively high difference between the R_2_Y and the Q_2_ (0.947 and 0.717, respectively).

Our next step was to run a univariate analysis, specifically, a *t*-test comparing the basal samples versus the final samples for each group independently. Then, the output was used to feed the mummichog algorithm [27] included in the MS-Peaks to pathways function embedded in MetaboAnalyst [28]. This function takes advantage of the high-resolution mass spectrometry data generated and provides some guidance for biological interpretation when fragmentation data is unavailable. Such an analysis revealed that the most relevant classes affected in the FOPLE group were glycerophosphocholines (PC), glycerophosphoserines (PS), oxidized glycerophosphoethanolamines (O-PE), glycerophosphoethanolamines (PE), 1-alkyl,2-acyl-PS, lysophosphatidylethanolamines (LPE), oxidized PS (O-PS), and dicarboxylic acids that showed an upward trend after the intervention (all *p* ≤ 0.05). In contrast, oxidized glycerophosphocholines (O-PC), phosphocholine ceramides (PC-Cer) or sphingomyelins (SM), phosphoethanolamine ceramides (PE-Cer), and ceramide-1-Phosphate (Cer-1-P) presented a downward trend after the intervention (all *p* ≤ 0.05). Besides, the algorithm could not provide relevant classes for the SOPLE group, possibly due to the low number of significant features. A detailed list of the statistical output from the algorithm and the compounds assignment is attached as Supplemental Material.

### 2.3. Differences after the Intervention in the General Biochemistry, Inflammatory Biomarkers, and FAs Profiles in Erythrocytes

Descriptive data from the general biochemistry, inflammatory biomarkers, and FA profiles in erythrocytes is presented in Table 3 and Table 4. The figures revealed that ApoA and HDL decreased after the SOPLE intervention whereas ApoB increased. While, in the FOPLE group, a significant increase in GGT and LDL in plasma (Table 2) and EPA, total PUFA ω-3 and Index ω-3 in erythrocytes were observed.

**Figure 2:**
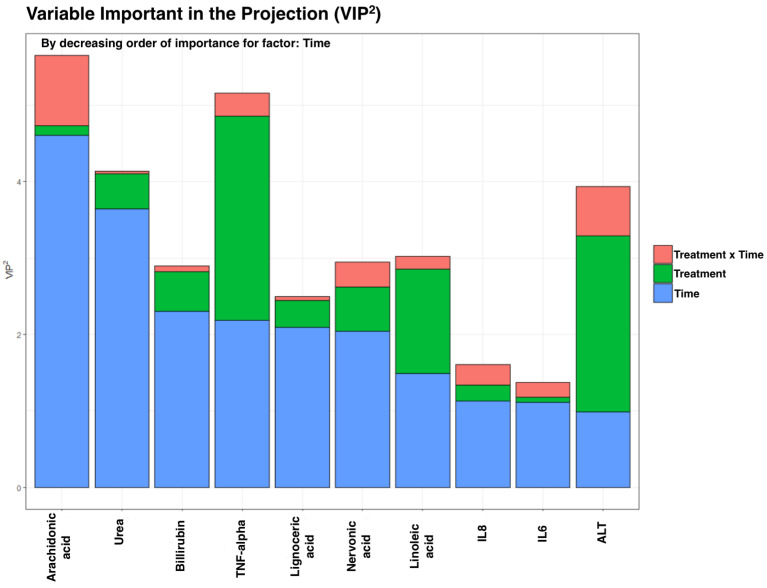
Most discriminant features according to the amOPLS approach for the integrated analysis of the general biochemistry, FAs, and inflammatory biomarkers in plasma. The variables are ordered by the VIP^2^ value corresponding to the time effect. VIP^2^: variable of importance in the projection. The higher it is, the more influence on the model.

As we have a low *n* and many variables, we analyzed using the AMOPLS approach. For such analysis, the variables analyzed in erythrocytes were treated as one block. We observed no significant main effects in samples from erythrocytes. Nevertheless, EPA and total ω-3 PUFA increased notably after the treatment in the FOPLE group, compared with the SOPLE group (*p* < 0.05). However, the results from plasma revealed a significant time effect (RSR *p*-value = 0.01) but not a treatment or interaction effect. Figure 2 shows the top 10 variables that discriminate samples ordered according to the variable of influence in the projection (VIP^2^) value associated with the time main effect. It is worth noting that one of the advantages of using AMOPLS is that we can obtain the influence of each variable on each factor, e.g., we observed that variables, such as arachidonic acid, tumor necrosis factor (TNF-α), and alanine-aminotransferase (ALT), have a high VIP^2^ in the time factor but also present a relevant influence on the treatment x time interaction, though this was not significant but need to be considered.

### 2.4. Data Integration Analysis for Biomarker Discovery Using Latent Components (DIABLO)

A Data Integration Analysis for Biomarker Discovery using Latent Components (DIABLO) was conducted in an exploratory manner due to the low number of samples. Data were divided into four blocks: lipidomics in plasma, plasmatic measurements (including FAs and inflammatory biomarkers), FAs in erythrocytes, and antioxidant enzymes and vitamins (data previously published in Baena et al.). Unfortunately, two subjects from the SOPLE group were dropped due to the lack of data corresponding to the antioxidant enzymes at one of the time points. The outcome was to identify the association between the different blocks of data and the difference between both groups, SOPLE and FOPLE. Samples from basal and final points from each group were merged.

DIABLO relies on identifying a limited number of correlated variables from multiple datasets to predict an outcome. In our study, the outcome was the «treatment». In brief, the method is an extension of sparse generalized canonical correlation analysis [29], which is a generalization of partial least squares for multiple matching datasets (*Q*) to a supervised learning framework [30,31]. The design matrix is a *Q* × *Q* matrix representing if and by how much each dataset should be correlated for the model’s algorithms in the DIABLO analysis. Values range from 0 to 1. For the present study, a value of 0.1 was utilized to pursue a maximum separation. Once the design matrix was assigned, a DIABLO model with two components was first fitted without any variable selection, and global performance was assessed using a 5-Mfold cross-validation. The model was tuned using internal functions of the mixOmics [31] package to find the optimal number of components and the optimal number of variables for each dataset. The main output of DIABLO is a set of components chosen in the model, a set of loading vectors, and a list of selected variables from each block of data associated with each component. Loadings are the coefficients assigned to each variable to define each component, and their absolute value represents the importance of each variable in DIABLO [30].

**Figure 3:**
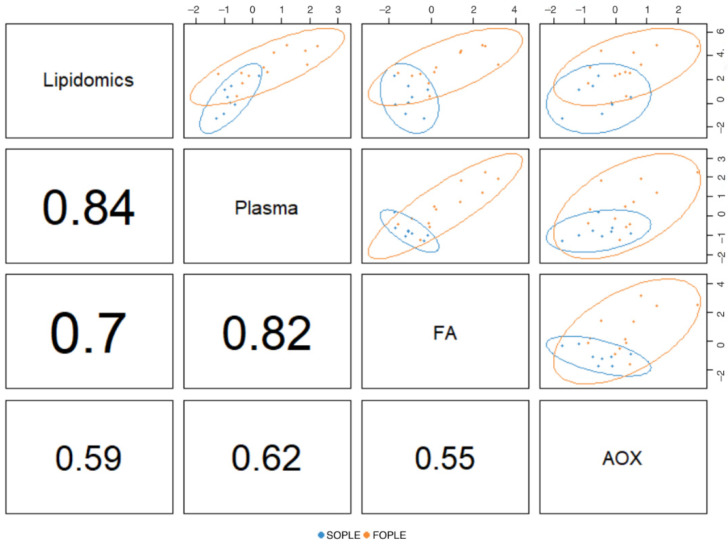
Correlation matrix corresponding to the unique component of the DIABLO model comparing the four data blocks. AOX: antioxidant enzyme activities, FA: fatty acids; plasma, measurements in plasma (including general biochemistry and FA). FOPLE: Fish-oil based formula; SOPLE: SO-based formula. The values presented correspond to the R^2^; a value closer to 1 reflects a better association between pairs.

The final optimized model included one component. Figure 3 presents a correlation matrix across the different data blocks for the first dimension. It is possible to observe the strong correlation, according to the high R^2^, between the lipidomics dataset and the block corresponding to the plasmatic FAs and general biochemistry parameters. The latter also presented a strong correlation with the block corresponding to the erythrocyte FAs. The correlation between the lipidomics and the FAs in erythrocytes is strong (r^2^ = 0.84). Finally, the association between the antioxidant enzymes and vitamins block appears less connected with the other data blocks.

**Figure 4:**
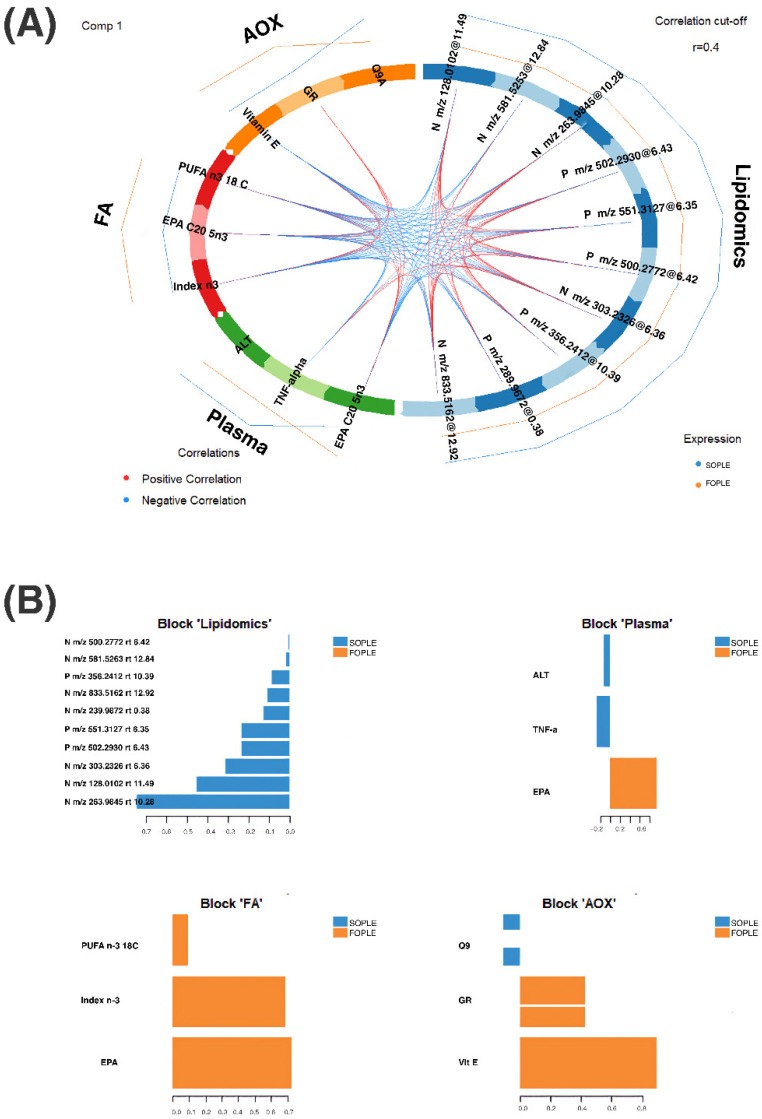
(**A**) Circos plot representing the connection between the different blocks of data and the correlation among the different variables in children after hematopoietic stem cell transplantation using two different parenteral nutrition lipid emulsions. ALT: alanine-aminotransferase; AOX: antioxidant block in plasma; EPA: eicosapentaenoic acid; FA: fatty acids block in erythrocytes; FOPLE: FO-based formula; GR: glutathione reductase; PUFA: polyunsaturated fatty acids; SOPLE: SO-based formula. The variables from the lipidomics block are labeled according to the ionization mode, positive or negative, the *m*/*z*, and the retention time. Pairs of variables were selected according to a correlation cut-off of r < 0.04. The different colors in the circle indicate the different data blocks, whereas the inner lines correspond to the correlation between pairs of variables. The outer lines represent the trend on each group for each variable. (**B**) The plot represents the loading weights of each elected variable on component one and for each data block. The color indicates the class where the variable has the maximum level as per the median. ALT: alanine-aminotransferase; AOX: antioxidant block in plasma; EPA: eicosapentaenoic acid; FA: fatty acids block in erythrocytes; FOPLE: FO-based formula; GR: glutathione reductase; PUFA: polyunsaturated fatty acids; SOPLE: SO-based formula. The variables from the lipidomics block are labeled according to the ionization mode, positive or negative, the *m*/*z*, and the retention time.

In addition, Figure 4A corresponds to a Circos diagram built on a similarity matrix [32] and represents the correlation between variables from different datasets. Pairs of variables presenting values above the cut-off (*r* = 0.4) are connected with red and blue inner lines according to the type of correlation (e.g., positive, or negative). We can observe that the main correlations include variables from all the data blocks. Remarkably, TNF-α is negatively correlated with EPA in plasma and EPA, the ω-3 Index, and total PUFA ω-3 in erythrocytes. Furthermore, we can observe a positive correlation between glutathione reductase and the ω-3 Index and EPA in plasma. The lipidomics block features are mostly correlated among them and connected with the other blocks, revealing that they might be FA-related metabolites as per their molecular weight.

Besides, Figure 4B represents the loading weights of the most relevant variables for each data block according to the optimized model. The component loading values highlight induced variables concerning the experimental factor treatment. 

## 3. Discussion

The present study shows that using a PN formulation based on ω-3 LC-PUFA in children undergoing HSCT for at least ten days can modulate the lipidome. The most relevant lipid classes affected in the enriched ω-3 LC-PUFA group were PC, PS, O-PE, O-PC, PE, SM, PE-Cer, Cer-1-P, O-PS, and di-carboxylic acids. However, we could not document relevant changes in those classes for the SOPLE group. In addition, the lipid profiles of erythrocytes were not affected by the PN lipid treatment, except for higher levels of EPA observed in the FOPLE group.

Intravenous lipid emulsions (IVLEs) are oil-in-water emulsions consisting of one or more triacylglycerol-containing oils, a mixture of phospholipid-based emulsifiers, and glycerol. In PN-admixtures, IVLEs are primarily used as a source of non-glucose energy and essential FAs (EFA). For clinical purposes, the FA profile is the most relevant characteristic of the IVLE. The available evidence demonstrates that ω-6 and ω-3 essential FAs and their long-chain metabolites can act through multiple mechanisms to promote the proliferation and differentiation of various stem cell types [33,34]. In particular, lipids have been found to participate in the mitochondrial activity to maintain HSC, a role previously overlooked due to HSC being thought of as primarily glycolytic. Moreover, there has been a re-emergence of how adipocytes in the bone marrow can regulate HSC [35]. In addition, metabolic disturbances can appear as procedure side effects of HSCT. Thus, HSCT survivors have an increased risk of abnormal lipid levels, and most patients continue showing abnormal levels at 1 and 2 years post-HSCT [36]. Indeed, HSCT patients require long-term follow-up, including lipid metabolism and thyroid function analysis. HSCT survivors demand the introduction of PUFAs into the diet to reduce the risk of developing lipid complications [37].

SO lipid emulsion may compromise immune function and promote hepatic damage due to its high proportion of PUFAs, i.e., LA and LNA phytosterols [38]. Combination lipid emulsions have been developed using medium-chain triglyceride oil, FO, and/or olive oil, providing adequate essential FAs and a minor concentration of PUFAs. Lipid emulsions containing FO offer potential advantages compared with traditional emulsions with a high SO content, such as decreased ω-6 and increased long-chain ω-3 PUFA concentrations, high concentrations of α-tocopherol, and reduced phytosterol content [39]. However, a systematic review and meta-analysis in children receiving PN have reported inadequate evidence that combination lipid emulsions offer any benefit regarding bilirubin levels, triglyceride levels, or incidence of infection compared with SO lipid emulsions [40]. Furthermore, a review from the Cochrane Collaboration has compared the safety and efficacy of all lipid emulsions for PN in preterm infants, including those with surgical conditions or PN-associated liver disease and cholestasis, using direct comparisons and pairwise meta-analyses. The authors did not find any particular lipid emulsion with or without FO to be better than another for growth, mortality, and prevention of liver diseases and other neonatal outcomes [41]. Nevertheless, long-term use of the composite FO lipid emulsion was well tolerated in PN-dependent children. The red blood cell composition of the FA profile alterations was consistent with the ω-3 PUFA-enriched composition of this emulsion without evidence of essential FA deficiency [42].

FA-based functional lipidomics investigate the structural and functional roles played by those compounds and their metabolites on in vivo changes due to metabolic or degradation pathways and provides the rationalization of these changes in connection with their biological significance. In the present study, we evidenced significant changes in lipids due to treatment and treatment *x* time interaction. However, we observed considerable variability in the AMOPLS statistical model (as the residuals accounted for >70% of the variability), which could be attributed mainly to the small number of patients included and followed-up. In the FOPLE group, the most affected lipid classes were phospholipids and oxidized phospholipids. This seems logical as the emulsion system for FOPLE uses relatively high amounts of soy lecithin and egg phospholipids, which include different proportions of PC; PE, phosphatidic acids (PA), N-acyl-PE, lyso-PE, and lyso-PC; sphingomyelins (SM) are also present in egg yolk phospholipids [43]. Indeed, the most significant PC species in the FOPLE group included LA, LNA, and other LC-PUFA of both ω-6 and ω-3 series (See Supplementary Tables S1–S3).

A cross-sectional study has reported the effects of two IVLEs with different FA profiles on the erythrocyte membrane lipidome in adult patients on home PN for more than two months in chronic intestinal failure. These authors documented lower percentages of arachidonic acid (20:4, ω-6) and higher EPA (20:5, ω-3) and DHA (22-6, ω-3) in erythrocytes of patients receiving and enriched FO emulsion; furthermore, they reported a higher estimation of delta-9 FA desaturase and lower of delta-5 FA desaturase activities in the FO-IVLE group than in control patients [44]. In addition, a previous report of our group showed that children receiving PN enriched in FO after HSCT increased their levels of plasma ω-3 LC-PUFA, particularly EPA [13]. In the case of red blood cell membranes, we observed only minor changes in the FA profiles for the FOPLE group, particularly for ω-3 LC-PUFA, with no significant changes in the estimation of the activity of FA desaturases. This appears concordant with the fact that the average life span of erythrocytes is about three months [45], and our patients received PN for about ten days, thus this was insufficient time to assess significant changes in the membrane of these cells.

Nevertheless, we observed significant correlations (R^2^ > 0.7) between the plasma and erythrocyte FA plasma profiles. It is worth noting that despite the short time of intervention, a clear correlation between EPA in erythrocytes and in plasma was observed. This association aids in confirming the effect of the intervention in the lipid profile of subjects. The measurement of the lipid profile only in plasma might be confusing as it can signal the presence of the IVLE in the blood stream. It is therefore the correlation that is a confirmation of the lipid integration in the cellular membrane.

Moreover, the DIABLO approach (Figure 3) also revealed strong correlations among the lipidomics dataset, plasma FA profiles, and erythrocyte FA profiles. In addition, we documented moderate correlations among those dimensions and antioxidant defense enzymatic activities (R^2^ > 0.5). It seems that an increase in the ω-3 LC-PUFA content in PN, as it occurs in the FOPLE emulsion, would result in an adaptive response of the body, increasing the activities of erythrocyte antioxidant enzymes to prevent further oxidation of membranes derived from LC-PUFA increase in plasma and erythrocytes.

Current evidence supports that ceramides, which are critical intermediates of sphingolipid metabolism, act as essential mediators of apoptotic cell death [46]. Several studies have demonstrated that cellular ceramide levels rise in response to various apoptotic stimuli, including some cytokines, as TNF-α [47], and irradiation [48] through activation of sphingomyelinases, stimulation of de novo ceramide synthesis, or both. Interventions that suppress ceramide accumulation indicate that ceramides are necessary and enough to trigger apoptosis [49]. Indeed, as the use of FOPLE emulsion in our study resulted in a significant decrease in PE-ceramides and ceramide-1-phosphate (see Supplementary Tables S1–S3), we hypothesize that the inclusion of FO in IVLE could contribute to regulating apoptosis in children after HSCT. Further studies are warranted to ascertain whether FO-enriched lipid emulsion can contribute to limiting apoptosis of transplanted cells and decrease pro-inflammatory and proapoptotic cytokines, such as TNF-α. On such regard, adipokines have multiple effects, including regulation of glucose metabolism, cell proliferation, inflammation, and angiogenesis. Concentrations of selected adipokines in children have been measured before and after HSCT, and it has been suggested that they could be good markers of disease burden and may influence metabolic complications of HSCT [50]. In the present study, we observed a significant inverse correlation between EPA in erythrocytes and plasma, and TNF-α.

aGvHD is a major factor limiting the successful outcomes of allogeneic hematopoietic cell transplantation (alloHSCT). A preliminary study in 51 patients with HSCT concluded that allotransplant recipients with aGvHD have an altered metabolic profile caused both by the disease and its immunosuppressive treatment [22]. Currently, few validated biomarkers can help predict the risk of aGvHD in clinical settings. An integrated metabolomics and transcriptomics study has suggested some biomarkers, especially five highly connected metabolites (lysophosphatidylcholine (LysoPC) [18:1], C16:0 PAF, LysoPC [P-16:0], LysoPC [18:2], and LysoPC [22:6]) that distinguish alloHSCT recipients with aGvHD from alloHSCT recipients without aGvHD in two separate cohorts [23]. Twenty-nine biomarkers were identified to distinguish between myelodysplastic syndrome (MDS), a neoplastic disease originating from HSCT patients, and healthy controls, mainly related to inflammation regulation and amino acid, FA, and energy metabolisms. To our knowledge, this is the first time where plasma metabolomics was combined with HSCT to study the pathogenesis and therapeutic target of MDS [51]. Some findings also support that the allogeneic immune response during aGvHD might be influenced by bile acids and the decreased production of aryl hydrocarbon receptor ligands by microbiota that could limit indoleamine 2,3-dioxygenase induction and influence allogeneic T cell reactivity [24]. In another study using a lipidomic approach, a pro-inflammatory metabolic profile was observed in adult patients who eventually developed aGvHD. Five potential pre-transplant biomarkers, 2-aminobutyric acid, 1-monopalmitin, diacylglycerols (DG 38:5, DG 38:6), and monounsaturated FA 20:1, demonstrated high sensitivity and specificity toward predicting post-transplant GvHD [25]. Another metabolomic study in adults with HSCT revealed ten differentially expressed plasma metabolites between participants with chronic GvHD and those without GvHD. The compounds were related to energy metabolism (*n* = 3), amino acid metabolism (*n* = 3), lipid metabolism (*n* = 2), caffeine metabolism (*n* = 1), and neurotransmission (*n* = 1), suggesting that chronic GvHD may be associated with expanded cellular energy and potentially mitochondrial dysfunction [26].

No studies have addressed the influence of PN lipid emulsion on aGvHD in children with HSCT, which warrants further investigation in this field.

### Strengths and Limitations of the Current Study

As far as we know, this is the first study in children with HSCT that evaluated changes of two different PN emulsions differing in their EFA and LC-PUFA content and distribution in plasma and erythrocyte lipids using a functional lipidomics approach. This study adds further support to the use of enriched ω-3 LC-PUFA in PN, with potential effects on prevention of cellular apoptosis and decrease in the pro-inflammatory status, associated with increased activity of main enzyme activities involved in the antioxidant defense status.

One of the main limitations of this study is the small sample size, which increased the risk of overfitting in our models. To avoid such overfitting, several cross-validation procedures were used, and the data were analyzed using different statistical methods to extract most of the data. Although the number of patients selected was relatively small, which is logical considering that these are children undergoing HSCT, an exploration of baseline data using PCA discarded the presence of outliers and demonstrated no differences between the SOPLE and FOPLE groups. Such analysis supported the homogeneity of the inclusion criteria used in the present study.

A major issue in untargeted lipidomics is the difficulty identifying compounds due to the absence of standards, which might lead to a dead end. However, the exploration using the approach suggested here provided at least some certainty at the subclass level. In such regard, the lack of fragmentation data from the lipidomics experiment was an explicit limitation of our study. However, we overcome this by using an advanced algorithm, namely mummichog, which let us perform a tentative annotation that provided the lipid class or subclass of the detected compounds, which changed over time. The advantage of using the most recent version of the mummichog algorithm relied on the retention time to improve the annotation and reduce the false-positive annotations, thus increasing the accuracy of the pathway assignment. We consider that such an approach is relevant because rather than identifying single compounds in lipidomics, identifying classes should tell us more about the patients’ metabolic status and the impact of the intervention.

## 4. Materials and Methods

### 4.1. Subjects

Seventeen children with HSCT requiring PN were consecutively identified and included in the present study. However, three children were excluded. Two of them because the PN was withdrawn before ten days marked as no longer required, and the third patient had severe blood sampling difficulties. Children were assigned to two different groups by the pharmacist in the hospital using a randomization program (SIGESMU^®^). A protocol standardized other medical interventions to maintain process uniformity during the study. Children were assigned to receive either FO-containing lipid emulsion [SO, medium-chain triacylglycerols (MCT), and FO]—ω-3 PUFA group, also named as FOPLE, or a standard SO formulation, also named as SOPLE, in PN [18]. Lipid emulsion containers were packed similarly to increase the effectiveness of blinding.

### 4.2. Inclusion and Exclusion Criteria

Children between 6 months and 14 years who were expected to receive PN for more than ten days after HSCT. Exclusion criteria were: (i) children <6 months or >14 y; (ii) clinical contraindications to PN; (iii) history of hypersensitivity to egg or soy proteins; (iv) severe organ failure or previous deranged liver function test (transaminase levels twice their average value and/or total bilirubin >2.5 mg/dL); and (v) requiring administration of a specialized lipid emulsion in PN. Parents or legal guardians provided written informed consent. The present randomized, double-blind controlled trial was approved by the Biomedical Ethical Committee at our institution and was registered in clinicaltrials.gov with the number NCT02199821.

### 4.3. Parenteral Nutrition

The requirements and composition of PN were very similar in all these patients and based on their energy expenditure estimated for each child by Schofield equation for weight and height [16]. AA intake in these patients was in a range of 1.7–3 g/kg/day, constituting between 12 and 16% of total caloric intake. Administered lipids constituted 35–40% of non-protein calories, and carbohydrates provided 60–65% of the remaining non-protein calories. The lipids were administered in separate perfusion from the rest of the nutrients in 20% solutions. Micronutrients were added in standard amounts of calcium, phosphorus, magnesium, water, and fat-soluble vitamins, zinc, copper, selenium, and chromium [17,18]. The mean dose for lipids in PN for all the patients was 1.5 g/kg/day up to 2 g/kg/day.

The lipid composition of each formula was as follows.

#### 4.3.1. Soybean Formula (SOPLE, 20 g of Purified SO per 100 mL)

The FA composition of the SOPLE emulsion was: LA 52%; LNA 8%; oleic acid (18:1n-9), 22%; palmitic acid (16:0), 13%; stearic acid (18:0), 4%; and other FAs, 1%. This emulsion contained 240 mg/L of tocopherol (10% is α-tocopherol). The other components were purified egg phospholipids, glycerol, and water for injections.

#### 4.3.2. FO-Containing Emulsion (FOPLE, 200 mg/mL (20%) of Triacylglycerols)

The composition of the FOPLE emulsion was 10 g of MCT, 8 g of SO, and 2 g of FO. Essential FA: LA, 25.72% (5.14 g/100 mL); LNA, 3.41% (0.68 g/100 mL); oleic acid, 13.44% (2.69 g/100 mL); EPA, 3.69% (0.74 g/100 mL); and DHA, 2.53% (0.51 g/100 mL), ratio ω-3/ω-6 1:2.7. This emulsion contained 190 mg/L of α-tocopherol. The other components were egg lecithin, glycerol, sodium oleate, ascorbylpalmitate, all-rac-alpha-tocopherol, sodium hydroxide, and water for injection.

Except for the lipid component, the PN bag was protected from ambient light using multilayered bags and a photo-resistant overwrap from the time of preparation to its administration. Afterward, opaque infusion systems were used during the infusion. Lipids were repackaged to adjust the prescribed dose for each patient under aseptic conditions in a horizontal laminar flow cabinet according to the protocols of the hospital pharmacy.

The number of days on total PN and on PN combined with enteral nutrition and the time of interruption of PN, i.e., when the patient tolerated appropriate amounts of enteral nutrition, were recorded. Enteral nutrition was started with minimum amounts of liquids with glucose or milk. ω-3 LC-PUFA were not included in this intake.

### 4.4. Sampling

Baseline blood samples were collected after a 12-h overnight fast at rest, lying, and using a PORT-A-CATH to draw a 3-mL sample in tubes containing EDTA. After centrifugation at 3500× *g* for 10 min, plasma was pipetted into Eppendorf tubes and frozen at −80 °C until analyzed.

### 4.5. Analysis of FA Profiles in Plasma and Erythrocytes

Blood samples were collected from patients from the antecubital vein into 6-mL blood collection tubes containing EDTA and processed within 2 h. After centrifugation at 3500× *g* for 10 min, plasma was divided into aliquots and frozen at −80 °C until the analysis. The cell pellet was immediately washed three times with a 0.9% NaCl isotonic solution, and the packed erythrocytes were collected and stored at −80 °C until lipid extraction.

Lipids from 0.2 mL plasma were extracted, and FA were transmethylated in a single step according to the Lepage and Roy methodology [52]. In brief, a direct methylation procedure was carried out in 5.0 mL of methanol-acetyl chloride 50:1 (*v*/*v*). To stop the reaction, 3 mL of 6.0% K_2_CO_3_ were added. After adding 150 µL of hexane, shaking, and centrifugation, the upper phase was separated and dried under nitrogen.

For the extraction of erythrocyte lipids, about 1 mL of washed cells were successively treated with 3 mL of isopropanol containing 50 mg/L butyl-hydroxytoluene as an antioxidant 2 mL of isopropanol, and 2mL of hexane. After centrifugation for 10 min at 3000× *g* at 4 °C, the upper phase of hexane was collected, and the infranatant was re-extracted three times with 2 mL hexane. The hexane extracts were combined and dried under nitrogen. For the methylation of erythrocyte FAs, we followed a similar process to that described above for plasma [52].

The methylated FAs were resuspended in 100 µL of hexane, and 1 µL injected into a Hewlett Packard HP5890 Series II chromatograph (Hewlett Packard, Palo Alto, CA, USA), with a capillary column (60 m × 32 mm inner diameter; 20 µm film thickness) impregnated with SP2330 FS (Supelco, Bellefonte, CA, USA). Running conditions were as described elsewhere [53]. FA methyl esters were identified by comparing retention times with previously run authentic standards and quantified using calibration curves (Sigma, St Louis, MO, USA).

### 4.6. Reverse Phase-Ultra Performance Liquid Chromatography-Fourier Transformation Mass Spectrometry (RP-UPLC-FTMS) Lipidomics

#### 4.6.1. Plasma Sample Preparation for RP-UPLC-FTMS Lipidomics

Plasma samples were vortexed for 5 s after thawing. Then, 100 µL of each sample was transferred to a 1.5-mL Eppendorf tube, and 500 µL of a mixed solvent (methanol: chloroform, 4:2, *v*/*v*) was added to each tube. The tubes were vortex mixed at 1600 relative centrifugal force (rcf) for 15 s and then sonicated in an ice-water bath for 5 min. The tubes were centrifuged in an Eppendorf 5420R centrifuge at 20,800× *g* and 10 °C for 15 min. A total of 500 µL of the supernatant was carefully taken out to another 1.5-mL tube, 300 µL of 50% aqueous methanol, and 200 µL of chloroform were added to each tube. The tubes were then vortex mixed at 1600 rcf for 30 s, followed by centrifugation in the same centrifuge at 20,800× *g* for six minutes to separate the whole phase into two phases, an upper aqueous phase (methanol-water) and a lower organic phase (chloroform). Using gel-loading tips, the organic phase was taken out to a 1-mL “V”-shape LC injection micro-vial. It was dried entirely at 30 °C in a nitrogen evaporator under a gentle nitrogen gas flow. The residue in each vial was dissolved in 100 µL of methanol. Two 15 µL aliquots of each solution were injected randomly to run RP-UPLC-FTMS to acquire two LC-MS datasets with positive-ion and negative-ion detection, respectively, in two respective rounds of the sample injections.

#### 4.6.2. Data Acquisition

For all UPLC-FTMS, a Dionex Ultimate 3000 UHPLC system coupled to a Thermo Scientific LTQ-Orbitrap Velos Pro mass spectrometer equipped with an electrospray ionization source was used.

RP-UPLC-FTMS runs were carried out for lipid analyses using a Waters BEH C8 UPLC column (2.1 × 50 mm, 1.7 μm) for chromatographic separation. The mobile phase was (A) 0.01% formic acid in water and (B) 0.01% formic acid in acetonitrile-isopropanol (1:1, *v*/*v*). The efficient gradient was 20% to 55% B in 5 min, 55% to 100% B in 12.5 min, and 100% B for 2.5 min before the column was equilibrated for 3.5 min at 20% B between injections. The column flow rate was 400 μL/min, and the column temperature was maintained at 60 °C. For relative quantitation, the MS instrument was operated in the survey-scan mode with full-mass FTMS detection at a mass resolution of 60,000 full-width at half maximum (FWHM) @ mass to charge ratio (*m*/*z*) 200. The mass scan range was *m*/*z* 80 to 1800 for both positive-ion and negative-ion detection.

#### 4.6.3. Data Preprocessing

Before handling the LC-MS datasets in positive and negative mode, each data file was converted to the ABF format. Then, converted data were processed with MS-DIAL v.4.36 [54] (each mode independently) for peak detection, retention time (RT) shift corrections, peak grouping, and peak alignment across all the samples (parameters are included as Supplementary Tables S4 and S5). The output of data processing is the pairs of MS *m*/*z*, LC RT (min), and the peak height of each detected metabolites or metabolite features across all the samples. Missing values were imputed in two rounds using the Random Forest [55] algorithm included in the “notame” package [56] available in R [57].

#### 4.6.4. Feature-Clustering

In untargeted metabolomics/lipidomics studies, several features can originate from the same metabolite, and thus, they are assumed to be highly correlated. Therefore, we have implemented the feature-clustering algorithm included in the notame [56] package within the data processing workflow. The algorithm identifies pairs of correlated features within a specified RT window and a correlation threshold (0.1 min and 0.90, respectively). The advantage of this process is that it facilitates the identification of correlated features and generates cleaner datasets, reducing the amount of noise that can disturb the subsequent multivariate analysis.

### 4.7. Multivariate Statistical Analysis

Multivariate exploratory analyses by PCA of the log-transformed and Pareto-scaled values were performed in SIMCA-P (version 15; Umetrics AB, Umeå, Sweden) to discard potential outliers, visualize the total variation of the metabolite profiles, and identify clustering patterns. For models containing one factor, i.e., time or treatment, an Orthogonal Partial Less Squares Discriminant Analysis (OPLS-DA) model was built to identify patterns or metabolic features discriminating between groups. The default seven-round cross-validation in the SIMCA software package was applied in these discriminant analyses. The cross-validation analysis of variance (CV-ANOVA) was calculated to assess the reliability, and a value ≤ 0.05 was considered significant. Moreover, the R_2_X and Q_2_ were evaluated to assess the robustness; in this case, values close to one reflect a reliable model.

For comparisons containing two factors, an AMOPLS was performed [58,59]. AMOPLS is a method that integrates the ANOVA-based submatrices associated with each effect and interaction into a single multiblock orthogonal PLS model. In such a way, we can detect any difference on each factor, even if it is subtle. This approach has shown to be very effective in distinguishing between multiple metabolic alterations and facilitating interpretation [58]. As with other standard factorial models, the interpretation can be made using score and loading plots and, in addition, the VIP^2^ values, which reflect the contribution of each variable to each main effect or interaction. Moreover, the statistical significance of each effect can be empirically estimated using a permutation test and thus evaluate the validity.

### 4.8. Univariate Statistical Analysis

#### T-Test

For lipidomics feature wise-analysis, a simple Welch’s *t*-test was performed comparing a pair of classes. Furthermore, the false discovery rate was used to correct for multiple testing and false positives, establishing a cut-off of q < 0.05 and obtaining the t.score for feeding the MS-Peaks to pathways analysis.

### 4.9. Pathway Analysis with Mummichog and Gene Set Enrichment Analysis (GSEA)

Pathway analysis with mummichog v2.06 [27] and GSEA [60] was developed using the MS-Peaks-to-Pathway module available on MetaboAnalyst v5.0 [28]. The input file required *m*/*z* values, RT values, *p*-values, t.score, and ionization mode (the mixed option that includes both negative and positive was selected). The molecular weight tolerance was set to 5 ppm and the option to enforce primary ions. Only features with at least one assigned primary ion [M + H]^+^, [M + Na]^+^, [M − H_2_O + H]^+^, [M − H]^−^, [M − H_2_O − H]^−^ and [M − 2H]^2−^ were valid. The *p*-value cut-off was set to α = 0.01 for the mummichog algorithm. We selected the meta-analysis option that combines the results from the GSEA and mummichog. The Lipids Sub-Chemical Class library includes 302 main lipid chemical classes for pathway analysis. Pathways including at least three entries and a combined *p*-value ≤ 0.05 were considered significantly modulated. It is important noting that in this version of the mummichog algorithm, the RT’s use will help increase the confidence and robustness of the potential compound matches. Retention is used to refine the grouping of signals into empirical compounds. Therefore, there is a reduction in the false-positive annotations and thus an increase in the accuracy of pathway activity prediction. Moreover, the use of the Lipids Sub-Chemical Class library narrows the spectrum of possibilities for annotation and matches with the analytical platform, which focuses on lipids.

## 5. Conclusions

In conclusion, this study confirmed that the FA profile of IVLE, particularly that enriched with ω-3 LC PUFAS, significantly influenced the plasma and erythrocyte functional lipidome. Our findings suggest that changes in specific lipid classes due to high levels of ω-3 LC-PUFA with increases in plasma PC, PE, PI, and PS and paralleled decreases in PE-ceramides and ceramide-1-phosphate may have an influence on several metabolic pathways and could contribute to improving the inflammatory status of children after HSCT.

## Figures and Tables

**Table 1:** Demographic characteristics and clinical complications in 10 children undergoing hematopoietic stem cell transplantation (HSCT) with two different lipid emulsions in parenteral nutrition, soybean oil, and n-3 PUFA-groups.

		SOPLE	FOPLE
Sex (male/female)		2/2	2/4
Age (months)		101.5 (8–180)	90.5 (31–132)
Pathology	Hematologic diseases	4	4
	Solid tumors		2
Type of HSCT	Allogeneic	4	4
	Autologous		2
GVHD		3	2
VOD		0	0
Time of engraftment	PMN: PMN > 500/mm^3^	15.5 (14–21)	13 (11–20)
	Platelets > 20,000	17.5 (15–24)	15 (12–68)
Total days of PN		13 (11–25)	16 (9–24)
Days of hospitalization		34 (31–37)	31 (29–43)

HSTC: hematopoietic stem cell transplantation; GvHD: graft-versus-host disease; PMN: Polymorphonuclear cell count; VOD: veno-occlusive disease. Data are expressed as median and the minimum and maximum range.

**Table 2:** AMOPLS output from the comparison between SO-based parenteral lipid emulsion (SOPLE) and FO-based parenteral lipid emulsion (FOPLE) before and after the intervention using treatment, time, and interaction as main effects using the lipidomics data.

Effect Name	RSS	RSR	RSR *p*-Value	R_2_Y *p*-Value	Tp1	Tp2	Tp3	To1
Treatment	0.08	1.159	0.03	0.03	0.046	0.042	0.825	0.239
Time	0.081	1.098	0.81	0.01	0.049	0.869	0.058	0.252
Treatment x Time	0.085	1.196	0.05	0.01	0.851	0.041	0.053	0.232
Residuals	0.753	1	NA	NA	0.054	0.049	0.064	0.277

NA: Not calculated; RSS: Relative Sum of Squares; RSR: Residual Structure Ratio; To: Orthogonal Component; Tp: Predictive Component.

**Table 3:** General biochemistry and inflammatory biomarker values in plasma from subjects in SO-based parenteral lipid emulsion (SOPLE) and FO-based parenteral lipid emulsion (FOPLE) formula groups before and after the intervention.

	SOPLE (*n* = 4)						FOPLE (*n* = 6)					
	Basal			Final			Basal			Final		
	Median	Min	Max	Median	Min	Max	Median	Min	Max	Median	Min	Max
Glucose (mg/dL)	90.5	63.0	103.0	94.0	77.0	208.0	94.5	68.0	103.0	91.5	82	120
Urea (mg/dL)	19.0	13.0	27.0	26.0	24.0	55.0	17.0	10.0	21.0	30.0	12	49
Creatinine (mg/dL)	0.40	0.30	0.53	0.43	0.35	0.68	0.48	0.35	0.55	0.42	0.33	0.52
AST (U/L)	44.0	19.0	114.0	41.5	15.0	81.0	36.0	17.0	82.0	32.5	25	71
ALT (U/L)	44.5	14.0	228.0	57.0	18.0	99.0	45.5	10.0	127.0	34.0	16	59
GGT (U/L)	19.0	9.0	46.0	36.0	19.0	161.0	28.5	17.0	77.0	102.0 ^a^	59	267
ALP (U/L)	105.5	87.0	220.0	144.5	117.0	332.0	155.5	102.0	182.0	160.5	106	332
ApoA (mg/dL)	98.5	71.0	105.0	56.0 ^a^	50.0	77.0	98.5	60.0	124.0	65.0	54	77
ApoB (mg/dL)	73.0	42.0	89.0	127.0 ^a^	53.0	145.0	97.5	54.0	205.0	120.5	88	222
Total cholesterol (mg/dL)	145	112	173	214	97	232	189	123	292	209	176	366
HDL (mg/dL)	35.0	26.0	42.0	15.0 ^a^	12.0	23.0	32.0	15.0	45.0	18.5	13	24
LDL (mg/dL)	13.5	9.0	19.0	110.5	49.0	181.0	15.5	0.0	38.0	131.0 ^a^	109	163
Triacylglycerols (mg/dL)	135	79	176	308	153	418	107	91	445	299	161	588
Bilirubin (mg/dL)	0.80	0.30	1.40	0.70	0.10	1.90	0.45	0.30	0.90	1.00 ^a^	0.5	1.7

Data presented correspond only to those patients that had the entire data blocks. ^a^ Significantly different in a *t-*test comparing basal vs. final time considering a *p*-value < 0.05 as the cut-off. ALT: alanine-aminotransferase; ALP: alkaline phosphatase; Apo: apolipoprotein; AST: aspartate aminotransferase; HDL: high-density cholesterol; GGT: gamma-glutamyl transferase; LDL: low-density cholesterol; FOPLE: FO-based formula; PCR: protein C-reactive; SOPLE: SO-based formula.

**Table 4:** Fatty acid profile of the red blood cell membrane from subjects in SO-based parenteral lipid emulsion (SOPLE) and FO-based parenteral lipid emulsion (FOPLE) formula groups before and after the intervention.

	SOPLE (*n* = 4)						FOPLE (*n* = 6)					
	Basal			Final			Basal			Final		
Fatty Acids, % Relative to Total FAs ^1^	Median	Min	Max	Median	Min	Max	Median	Min	Max	Median	Min	Max
Myristic acid (C14:0)	0.80	0.44	1.76	0.66	0.49	1.10	0.53	0.36	3.03	0.63	0.37	0.96
Palmitic acid (C16:0)	24.70	23.60	26.10	24.10	22.10	25.0	23.30	22.70	28.30	23.75	22.60	25.50
Palmitoleic acid (C16:1)	0.49	0.37	0.70	0.19	0.00	0.63	0.39	0.00	1.18	0.25	0.00	0.83
Margaric acid (C17:0)	0.39	0.33	0.60	0.87 ^a^	0.45	0.98	0.39	0.00	1.27	0.16	0.00	0.90
Estearic acid (C18:0)	15.85	15.70	17.00	15.80	15.30	20.3	15.95	13.00	17.30	15.70	14.90	17.70
Oleic acid (C18:1n-9c)	15.35	13.00	20.50	15.30	14.40	16.2	14.85	13.70	25.20	15.15	14.60	17.10
Vaccenic acid (C18:1n-7)	0.99	0.50	1.17	1.09	0.92	1.25	1.11	0.95	1.49	1.11	1.01	1.21
Linoleic acid (C18:2ω-6)	7.90	7.70	8.50	8.85	7.70	10.5	9.30	5.40	9.50	8.15	7.30	9.90
Arachidic acid (C20:0)	0.42	0.00	0.48	0.23	0.00	0.50	0.00	0.00	0.53	0.43	0.00	0.50
Linolenic acid (C18:3ω-3)	0.16	0.00	0.41	0.00	0.00	0.33	0.00	0.00	0.40	0.00	0.00	0.00
Behenic acid (C22:0)	1.63	1.21	1.86	1.59	1.54	1.72	1.90	1.17	3.97	1.72	1.55	1.94
Dihomo-γ-linolenic acid (C20:3ω-6)	1.78	1.69	1.95	1.69	1.51	1.93	1.41	0.00	2.39	1.33	1.16	2.21
Dihomo-α-linolenic (C20:3ω-3)	0.61	0.57	0.73	0.75	0.62	1.52	0.54	0.42	0.85	0.66	0.38	1.10
Arachidonic acid (C20:4ω-6)	14.80	10.30	17.60	14.45	13.10	15.8	14.65	9.70	16.40	13.65	12.80	15.10
Eicosapentanoic acid (C20:5ω-3)	0.00	0.00	0.34	0.00	0.00	0.57	0.35	0.00	0.46	1.51 ^a^	0.46	2.12
Lingnoceric (24:0)	4.65	3.63	5.07	4.44	4.36	4.89	4.59	2.88	5.25	4.50	4.15	4.70
Nervonic acid (24:1n9)	3.76	3.06	5.14	3.69	2.93	4.47	3.47	2.08	6.11	3.25	2.95	3.88
Docosapentanoic acid (C22:5ω-3)	1.33	1.28	1.78	1.43	1.24	1.53	1.48	0.92	1.94	1.84	1.38	2.41
Docosahexaenoic acid (C22:6ω-3)	3.56	2.44	4.10	3.73	2.37	4.04	4.05	2.74	4.93	4.43	4.07	6.15
SFA	48.80	47.20	49.90	48.55	47.10	49.9	47.90	46.30	49.00	47.05	46.10	48.50
UFA	51.20	50.10	52.80	51.45	50.10	52.90	52.10	51.00	53.70	52.95	51.50	53.90
MUFA	20.65	19.50	24.70	20.15	19.40	21.60	19.80	18.70	29.90	19.65	19.50	22.10
SFA/MUFA ratio	2.35	2.00	2.50	2.45	2.20	2.50	2.40	1.60	2.60	2.40	2.20	2.50
DUFA	7.90	7.70	8.50	8.85	7.70	10.5	9.30	5.40	9.50	8.15	7.30	9.90
MUFA/DUFA ratio	2.65	2.50	2.90	2.30	1.90	2.8	2.30	2.00	5.60	2.40	2.10	2.80
PUFA	30.50	26.60	32.10	31.20	30.30	31.9	32.40	21.40	32.80	32.70	30.20	34.00
MUFA/PUFA ratio	0.65	0.60	0.90	0.70	0.60	0.70	0.60	0.60	1.40	0.60	0.60	0.70
PUFA ω-6	24.40	20.80	27.10	25.20	24.50	25.6	25.35	16.00	25.90	23.45	22.90	25.80
PUFA ω-3	5.80	4.40	6.90	6.15	5.10	6.60	6.65	4.70	7.80	8.70 ^a^	7.10	10.70
PUFA ω-6 >18 C	16.55	12.30	19.40	16.25	14.70	17.5	16.35	10.70	17.80	15.35	14.00	16.60
PUFA ω-3 >18 C	5.45	4.40	6.90	5.95	5.10	6.60	6.55	4.70	7.80	8.70 ^a^	7.10	10.70
UI	2.65	2.40	3.00	2.70	2.60	2.90	2.85	2.30	3.10	3.00	2.80	3.30
Ratio ω-6/ω-3	3.84	3.62	6.13	4.14	3.70	4.91	3.58	2.99	5.49	2.68	2.19	3.35
Index ω-3	3.56	2.44	4.44	3.87	2.37	4.34	4.44	2.74	5.36	6.14 ^a^	4.67	7.70
Delta9 desaturase	0.97	0.76	1.30	0.99	0.71	1.02	0.92	0.82	1.94	0.99	0.83	1.11
Delta6 desaturase	0.23	0.22	0.23	0.18	0.17	0.25	0.18	0.00	0.26	0.17	0.12	0.27
Delta5 desaturase	0.12	0.10	0.19	0.12	0.10	0.13	0.10	0.00	0.17	0.10	0.08	0.17

Data presented correspond only to those patients that had the entire data blocks. ^1^ The amount of each fatty acid was calculated as a percentage of the total fatty acid content (relative%). ^a^ Significantly different in a *t*-test comparing basal vs. final time considering a *p*-value of < 0.05 as the cut-off. DUFA: double unsaturated fatty acid; FOPLE: FO-based formula; MUFA: monounsaturated fatty acid; PUFA: polyunsaturated fatty acid; SFA: saturated fatty acid; SOPLE: SO-based formula; UFA: unsaturated fatty acid, UI: unsaturation index.

## Data Availability

Data not presented in the manuscript or supplementary material can be provided if available.

## Supplementary Materials

The following supporting information can be downloaded at: https://www.mdpi.com/article/10.3390/ijms23073667/s1.
