# Supplementary material for: The Metabolic Impact of Two Different Parenteral Nutrition Lipid Emulsions in Children after Hematopoietic Stem Cell Transplantation: A Lipidomics Investigation
Source: Int J Mol Sci. 2022 Mar 27;23(7):3667. doi: 10.3390/ijms23073667 (PMC8998446; doi:10.3390/ijms23073667)
Supplement: Supplementary file 1 [file ijms-23-03667-s001.zip › comite investigacion_NP.pdf]

Servicio Andaluz de Salud
CONSEJERÍA DE SALUD

Hospital Universitario Reina Sofía

Óscar Degayón Rojo, Secretario de la Comisión de Ética e Investigación Sanitaria del Hospital Universitario Reina Sofía de Córdoba,

CERTIFICA

Que en la reunión de la Subcomisión Permanente de Investigación Sanitaria celebrada el día 18 de Marzo de 2010 se ha estudiado y evaluado el Proyecto de Investigación titulado "Efecos metabólicos y en sistema inflamatorio y antioxidante de diferentes emulsiones lipídicas en nutrición parenteral en niños críticos", en el que figura como Investigadora Principal la facultativo Dª Mercedes Gil Campos, adscrita al Servicio Críticos y Urgencias Pediátricos, habiendo considerado los integrantes de dicha Subcomisión que el citado proyecto respeta los principios fundamentales establecidos en la Declaración de Helsinki de 1964, de la Asociación Médica Mundial, y enmiendas posteriores, y en el Convenio del Consejo de Europa de 1996, relativo a los Derechos Humanos y a la Biomedicina, y se adecua a las condiciones de la convocatoria, demostrando sus autores conocer suficientemente los antecedentes y el estado actual del tema que proponen investigar, estando bien definidos sus objetivos y siendo adecuada su metodología, por lo que hacen constar la viabilidad en todos sus términos del proyecto de investigación, estimando que los resultados pueden ser de gran interés.

Se hace constar, de acuerdo con el art. 27,5 de la Ley 30/1992, de 26 de noviembre, de Régimen Jurídico de las Administraciones Públicas y del Procedimiento Administrativo Común, que la presente certificación se emite con anterioridad a la aprobación del Acta correspondiente.

En Córdoba, a dieciocho de Marzo de dos mil diez.

EL SECRETARIO

COMISIÓN DE ÉTICA E INVESTIGACIÓN SANITARIAS
Subcomisión de Investigación
HOSPITAL UNIVERSITARIO REINA SOFÍA
CÓRDOBA

Fdo.: Óscar Degayón Rojo
