# Supplementary figures and images for: The Metabolic Impact of Two Different Parenteral Nutrition Lipid Emulsions in Children after Hematopoietic Stem Cell Transplantation: A Lipidomics Investigation
Source: Int J Mol Sci. 2022 Mar 27;23(7):3667. doi: 10.3390/ijms23073667 (PMC8998446; doi:10.3390/ijms23073667)

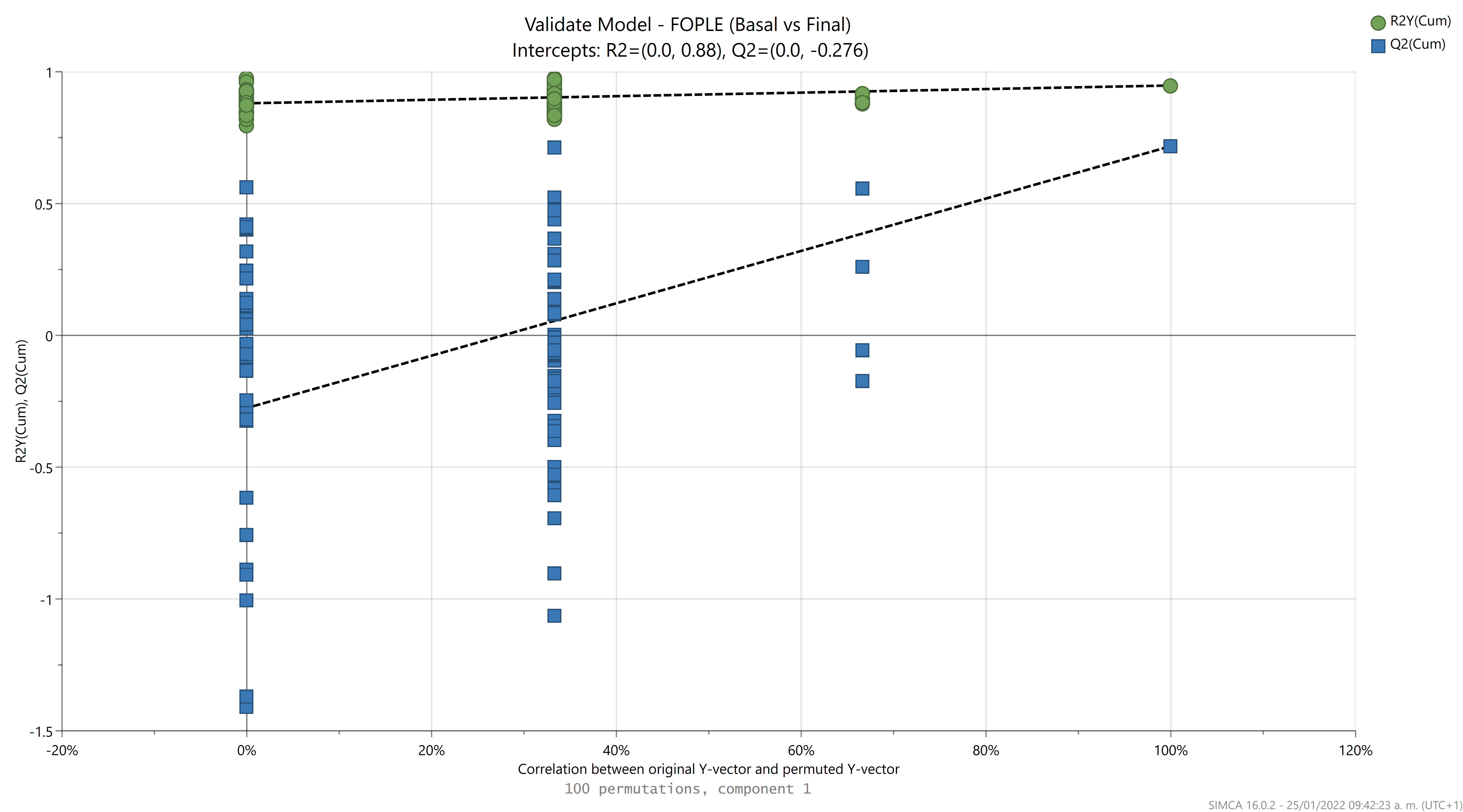

Supplement: Supplementary file 1 [file ijms-23-03667-s001.zip › Supplementary Figure S1.tif]
